# Correction: World Congress on Osteoporosis, Osteoarthritis and Musculoskeletal Diseases (WCO-IOF-ESCEO 2024)

**DOI:** 10.1007/s40520-024-02889-2

**Published:** 2024-12-06

**Correction: Aging Clin Exp Res (2024) 36:174** 10.1007/s40520-024-02766-y

In this article the author’s name S. Gunsenkhorloo was incorrectly written as S. S. Surenkhorloo in the article abstract P662.

The original article has been corrected.

